# Gadoxetic acid-enhanced MRI of hepatocellular carcinoma: Diagnostic performance of category-adjusted LR-5 using modified criteria

**DOI:** 10.1371/journal.pone.0242344

**Published:** 2020-11-13

**Authors:** Jae Hyon Park, Yong Eun Chung, Nieun Seo, Jin-Young Choi, Mi-Suk Park, Myeong-Jin Kim

**Affiliations:** Department of Radiology, Yonsei University College of Medicine, Seoul, Republic of Korea; Medical University of Vienna, AUSTRIA

## Abstract

The Liver Imaging Reporting and Data System (LI-RADS) is widely adopted for the noninvasive diagnosis of hepatocellular carcinoma (HCC). Herein, possible strategies to improve the diagnostic performance of LR-5 without reducing specificity for HCC were investigated. This retrospective study included 792 patients who underwent gadoxetate disodium-enhanced magnetic resonance imaging. Hepatic observations were categorized according to LI-RADS v2018 and categories were readjusted by upgrading LR4 to LR5 using ancillary features, arterial phase hyperenhancement (APHE) interpreted with subtraction images, indication of no washout when APHE was absent, extension of washout to the transitional phase, and subthreshold growth as a major feature. Based on LI-RADS v2018, LR-5 showed a sensitivity of 71.9% and a specificity of 97.9% for the diagnosis of HCC. Category-readjusted LR-5 after upgrading LR-4 to LR-5 using ancillary features favoring HCC in particular, subthreshold growth as a major feature, extending washout to transitional phase and APHE interpreted using subtraction images showed significantly increased sensitivity (*P*<0.001) without decreased specificity (*Ps*>0.05). The sensitivity of LR-5 can be improved without loss of specificity via category readjustment using AFs favoring HCC in particular, subthreshold growth as a major feature, extending washout to transitional phase and APHE interpreted with subtraction images.

## Introduction

Hepatocellular carcinoma (HCC) is one of the few malignancies that can be diagnosed noninvasively without biopsy in patients with cirrhosis or chronic liver disease, largely due to its unique vascular pattern of arterial hyperenhancement followed by washout [1]. Current clinical guidelines suggest that a hepatic observation larger than 1cm can be diagnosed as HCC with high specificity using either dynamic computed tomography (CT) or magnetic resonance imaging (MRI) in high risk patients [1, 2] but until the Liver Imaging Reporting and Data System (LI-RADS) was first released in 2011 by the American College of Radiology, considerable variations in image interpretation and reporting impeded the correct diagnosis of HCC [3].

Nowadays, LI-RADS is widely accepted as a good scheme for interpreting and reporting imaging features of hepatic observations on dynamic CT and MRI in patients at high risk of HCC, with hepatic lesion being categorized from LR-1 (definitely benign) to LR-5 (definitely HCC) [4–6]. LI-RADS version 2018 (v2018) [7] is the fourth update of this system, and important changes have been made compared to LI-RADS version 2017 (v2017) [7] to achieve simplicity and consistency with the American Association for the Study of Liver Disease (AASLD) 2018 clinical practice guidance for HCC [8] and the Organ Procurement and Transplantation Network (OPTN) classification system [7, 9]. In a subsequent study, however, LR-5 observations according to LI-RADS v2018 [7] showed increased sensitivity (81% vs. 68%) but reduced specificity (94% vs. 99%) for HCC [6] compared to LR-5 observations according to LI-RADS v2017. In addition, while improved, some elements of the diagnostic algorithm in LI-RADS v2018 remain controversial and in need of validation.

The aim of our study was, thus, to investigate the diagnostic performances of adjusted LR-5 for HCC when LR-4 is upgraded to LR-5 using ancillary feature (AF), interpreting nonrim-arterial phase hyperenhancement (APHE) in arterial subtraction images, extending washout to transitional phase, and considering no washout when APHE is absent, and compare them to the diagnostic performance of the original LR-5. In addition, we also evaluated the diagnostic performance of LR-5 adjusted with subthreshold growth being regarded as a major feature.

## Materials and methods

### Patients

This Health Insurance Portability and Accountability Act-compliant (HIPAA) study was approved by our institutional review board of Yonsei University College of Medicine and written informed consent was waived due to its retrospective study design. Using electronic medical records, patients with cirrhosis or chronic hepatitis B virus infection who underwent gadoxetate disodium-enhanced MRI between January 2009 and December 2014 for the evaluation of focal hepatic lesions were identified. Herein, a hepatic observation was defined as any area distinct from the background liver detected on any phase of routine MRI sequences [7]. Inclusion criteria were patients who (1) underwent liver surgery within 6 months from the date of the MRI exam, (2) had no history of treatment for hepatic observations before the MRI exam and (3) were pathologically diagnosed such as through surgical resection. On the other hand, patients who (1) had underlying congestive hepatopathy or iron-deposition liver disease including hemochromatosis or Wilson’s disease, (2) had >3 hepatic observations and (3) did not have all the required images of the MRI protocol were excluded from analysis. For patients with more than one but <3 hepatic observations, the largest observation with a corresponding histopathologic diagnosis was analyzed.

### MRI acquisition

All patients underwent MRI examinations on a 3.0- MRI unit. Dynamic MRI studies of the liver were performed after 10 mL of gadoxetate disodium (Primovist; Bayer AG, Berlin, Germany) was injected followed by 20 mL of 0.9% saline at injection rate of 1 mL/s. T1-weighted 3D gradient-echo imaging was obtained before contrast injection. Arterial phase imaging was initiated using either a test bolus technique with 1mL of gadoxetic acid or the bolus-tracking technique, and images from the portal venous phase, transitional phase, and hepatobiliary phase were obtained at approximately 60, 90, and 150 seconds and 20 minutes after the administration of the contrast agent began, respectively. Subtraction images were automatically generated after image acquisition on the MRI console that provided image-by-image subtractions or were manually generated by AquariusNET (Tera-Recon, San Mateo, CA, USA) between the unenhanced and arterial phases of each patient.

Other MRI sequences included an axial dual-echo T1-weighted breath-hold gradient echo sequence for acquisition of in-phase and out-of-phase images, an axial respiratory-triggered turbo spin-echo T2-weighted sequence with fat saturation, an axial half-Fourier acquisition single-shot turbo spin-echo T2-weighted sequence with fat saturation, and diffusion-weighted imaging with respiratory-triggered single-shot echo planar imaging sequences with *b* values of 0, 50, 400 and 800 sec/mm^2^ or 50, 400 and 800 sec/mm^2^.

### MR image analysis and LI-RADS category assignment

Two board-certified radiologists with 11 years (Y.E.C) and 15 years (J.Y.C) of experience with liver MRI retrospectively reviewed and analyzed the images together. Prior to image analysis, another radiologist (J.H.P) selected a lesion (the largest lesion, if multiple lesions in a patient had received histopathologic diagnoses) corresponding to the pathology report findings. All MRIs were reviewed via a picture archiving and communication system (PACS) (Centricity Radiology RA 1000; GE Healthcare, Chicago, IL, USA). While both readers were aware that all patients had undergone MRI because of suspected focal hepatic lesions and that the patients either had liver cirrhosis or chronic hepatitis B virus infection, the readers were blinded to the histopathological results.

Lesion size, location, major features, targetoid mass features and AFs as well as the final LR-category of the hepatic observations were evaluated according to LI-RADS v2018 [7]. Presence of APHE was examined in both the ordinary late arterial phase image and arterial subtraction image. A minimum interval of two weeks had to pass before the arterial subtraction images were analyzed to avoid possible recall bias.

### Validation study of the diagnostic performances of different category-adjusted LR-5 for HCC

Diagnostic performances of different category-adjusted LR-5 for HCC were compared to the diagnostic performance of the original LR-5. Categories were readjusted under six different conditions: 1) using AFs favoring malignancy in general including AFs favoring HCC in particular (any one of subthreshold growth, restricted diffusion, mild-moderate T2 hyperintensity, corona enhancement, fat sparing in solid mass, iron sparing in solid mass, transitional phase hypointensity and hepatobiliary phase hypointensity, nonenhancing capsule, nodule-in-nodule, mosaic architecture, blood products in mass, fat in mass more than adjacent liver) to upgrade LR-4 to LR-5; 2) using AFs favoring HCC in particular (any one of nonenhancing capsule, nodule-in-nodule, mosaic architecture, blood products in mass, fat in mass more than adjacent liver) to upgrade LR-4 to LR-5. For conditions 1) and 2), categories were adjusted in the presence of ≥1 AF favoring malignancy in general (including AFs favoring HCC in particular) and in the presence of ≥1 AF favoring HCC in particular only, respectively, even though upgrade from LR4 to LR5 is prohibited in LIRAD v2018. No category adjustment was made in the presence of ≥1 AF favoring benignity which was consistent with the v2018 diagnostic algorthm. In addition, LR-3 lesions that had already been upgraded to LR-4 lesions were not eligible for category adjustment using AFs; 3) LI-RADS v2018 [7] dictates that nonrim APHE can only be called if the signal intensity of the observation on the arterial phase is unequivocally greater than the liver and states that the subtraction image may be used optionally when evaluating APHE. Under condition 3), APHE is called if hyperintensity is seen in the subtraction image, which is made by the subtraction of pre-contrast image from late arterial phase image; 4) In addition, LI-RADS v2018 [7] defines washout as any temporal reduction in enhancement relative to composite liver tissue in the portal venous phase regardless of the presence of APHE in the late arterial phase. Under condition 4), presence of washout was interpreted only when there was an initial “wash-in” or APHE in the late arterial phase. All other reduced enhancements in the portal venous phase of hepatic observations without APHE in the late arterial phase were not considered as washout; 5) Extending washout to the transitional phase; and 6) Considering subthreshold growth as a major feature rather than AFs favoring malignancy: in previous LI-RADS v2017, threshold growth was defined as one of “≥ 50% size increase within ≤6 months”, “≥ 100% size increase within > 6 months” and “new ≥ 10mm nodule within ≤ 24 months” wherein the last two definitions no longer meet criteria for threshold growth and are considered as subthreshold growth in LI-RADS v2018 while the definition of subthreshold growth being “unequivocal size increase of mass, less than threshold growth” remains unchanged in both LI-RADS v2017 and LI-RADS v2018. In this study, the diagnostic performance was evaluated when “≥ 100% size increase within > 6 months” and “new ≥ 10mm nodule within ≤ 24 months” were again considered as threshold growth as in previous LI-RADS v2017 rather than using LI-RADS v2018 definition.

### Histopathologic diagnosis

Diagnosis of HCC and non-HCC malignancies were confirmed via pathology. Benign diagnoses were obtained through pathology (n = 3) or typical imaging features or stability at imaging for at least 2 years (n = 226). The fibrosis stage of the liver parenchyma was assessed according to the Batts-Ludwig scoring system from F0, no fibrosis to F4, cirrhosis [10, 11], if available in the pathology report.

### Statistical analysis

Patient characteristics were compared between the two groups using the *X*^2^-test or the Fisher exact test for categorical variables and the Student *t* test for continuous variables. Since only one lesion was selected for image analysis, the endpoints were analyzed on a per patient basis [5]. Estimates and 95% confidence intervals (CIs) of diagnostic performance including sensitivity, specificity, positive predictive value and negative predictive value were calculated for LR-5 as well as for the combination of LR-4 and LR-5 in LI-RADS v2018. Diagnostic performances of the category-adjusted LR-5 were also calculated and compared to that of the original v2018 LR-5 using McNemar’s test. Receiver operating characteristic (ROC) curves were drawn and an area under the curve (AUC) was calculated. Pairwise comparison of ROC curves were done and *P*-values were recorded. A two-sided *P*-value <0.05 was considered to indicate a statistically significant difference. All statistical analyses were performed using MedCalc, version 19.0.7 (MedCalc Software, Ostend, Belgium) and SPSS, version 25 (IBM, Chicago, IL, USA).

## Results

### Patient characteristics and pathologic findings

**Fig 1:**
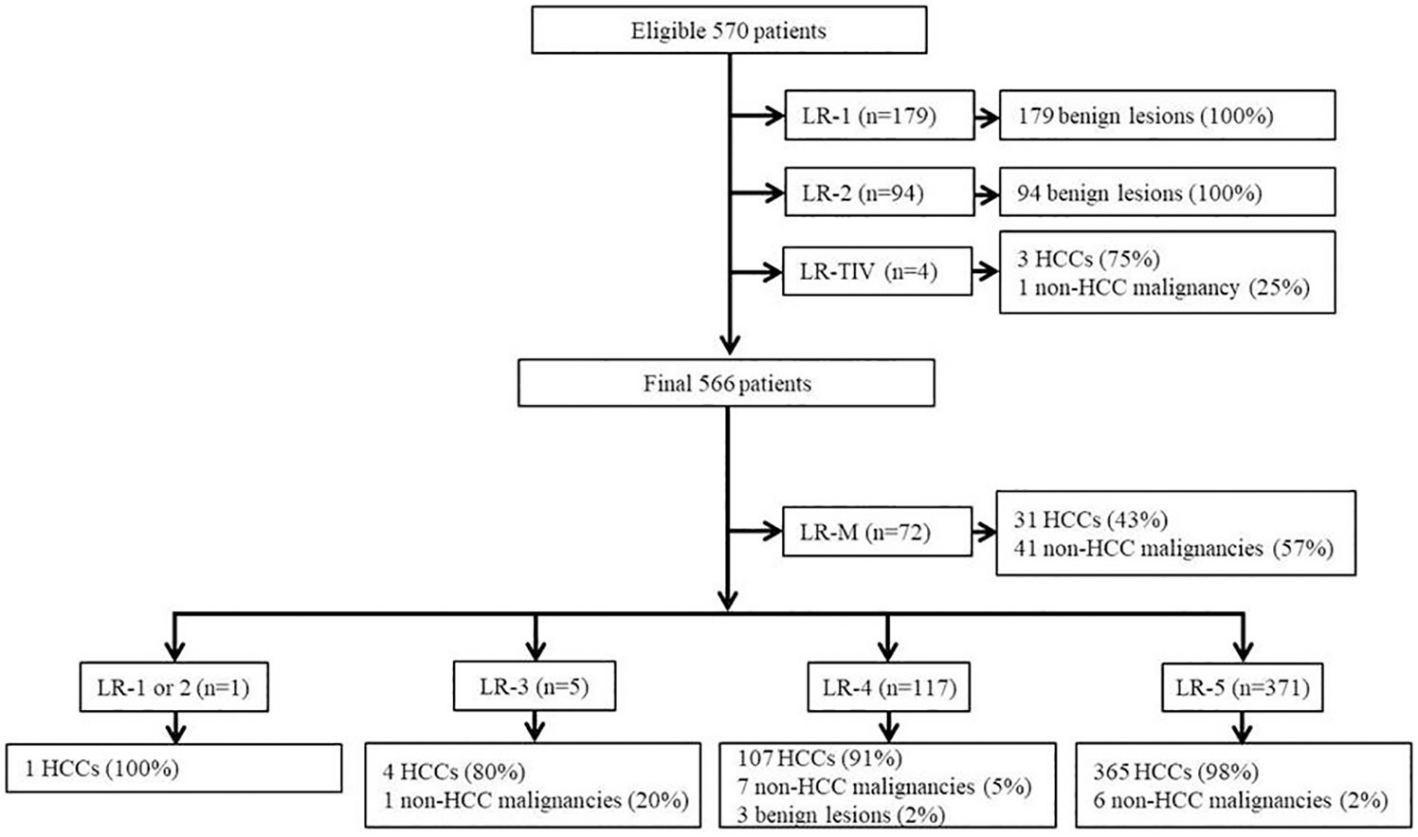
Study flow diagram.

Based on the inclusion and exclusion criteria, 796 potential eligible patients were identified. After excluding four patients with tumor in a vein, 792 patients were finally included in this study (Fig 1). Clinico-pathologic characteristics of the 792 patients (616 men and 176 women; mean age, 56 years±10; range, 28–85 years) are summarized in Table 1. Median size of HCC, non-HCC malignancies and benign lesions were 29.4mm, 36.2mm and 11.0mm, respectively. Out of the total 508 HCCs, 5 were <10mm, 90 were 10-19mm and 413 were ≥20mm in size.

**Table 1:** Clinical-pathologic characteristics of patients and hepatic observations.

Characteristics	Value
Patients (n = 792)	
Mean age (y)*	56.2±10.0
Sex	
Men	616 (77.8)
Women	176 (22.2)
Cause of liver disease	
Hepatitis B virus	650 (82.1)
Alcohol	51 (6.4)
NASH	43 (5.4)
Hepatitis C virus	27 (2.4)
Autoimmune	1 (0.1)
Cryptogenic	20 (2.5)
Number of observations per patient	
1	666 (84.1)
2	65 (8.2)
3	61 (7.7)
Lesions (n = 792)	
Median size (mm)**	25.2 (25.0)
HCC	29.4 (20.5)
Non-HCC malignancies	36.2 (26.4)
Benign lesions	11.0 (9.0)
Final diagnosis	
HCC	508 (64.1)
Non-HCC malignancies	55 (6.9)
IMCC	27 (49.1)
cHCC-CCA	23 (41.8)
Metastasis	4 (7.3)
Sarcomatoid cHCC-CCA	1 (1.8)
Benign lesions (n = 229)	
Hemangioma	143 (62.4)
Dysplastic or regenerative nodules	46 (20.1)
FNH-like nodule	23 (10.0)
Eosinophilic infiltration	12 (5.2)
Focal fat-deposition	3 (1.3)
Inflammatory pseudotumor	1 (0.4)
Focal fat-sparing	1 (0.4)
Acute and chronic inflammation with granulation tissue and fibrosis	1 (0.4)
Pathologically confirmed liver fibrosis (n = 579)	
Cirrhosis (Grade 4)	332 (58.7)
Septal fibrosis (Grade 3)	99 (17.5)
Periportal fibrosis (Grade 2)	80 (14.1)
Portal fibrosis (Grade 1)	55 (9.7)
Median time interval between MRI and pathologic diagnosis (d)**	13 (14)

Note- Unless stated otherwise, data are number of patients or observations. Data in parentheses are percentages.

Abbreviations: cHCC-CCA, combined HCC-choangiocarcinoma; FNH, focal nodular hyperplasia; HCC, hepatocellularcarcinoma; IMCC, intrahepatic mass-forming cholangiocarcinoma; y, years; d, days

*Data are means ± standard deviations.

**Data are presented as median values. Data in parentheses are interquartile ranges and were calculated as the difference between the 75th and 25th percentiles.

### Diagnostic performance for HCC using LI-RADS v2018

Based on the diagnostic algorithm of LI-RADS v2018, the final LI-RADS categories of the 792 hepatic observations were as follows: 73 LR-M, 116 LR-1, 52 LR-2, 63 LR-3, 118 LR-4, and 370 LR-5 (Fig 1). Based on these categorizations, LR-5 showed a sensitivity of 71.9% and a specificity of 97.9% for the diagnosis of HCC (Table 2).

**Table 2:** Sensitivity, specificity, accuracy, positive predictive value (PPV) and negative predictive value (NPV) of hepatocellular carcinoma (HCC) under various categorizations via LI-RADS v2018.

	Sensitivity (%)	Specificity (%)	PPV (%)	NPV (%)	Accuracy (%)	*P*-value^a^	*P*-value^b^
LIRADS v2018 LR-4 and 5	92.9 (472/508) [90.3, 95.0]	94.4 (268/284) [91.0, 96.8]	96.7 (472/488) [94.8, 97.9]	88.2 (268/304) [84.4, 91.1]	93.4 (740/792) [91.4, 95.1]	-	-
LIRADS v2018 LR-5*	71.9 (365/508) [67.7, 75.7]	97.9 (278/284) [95.5, 99.2]	98.4 (365/371) [96.5, 99.3]	66.0 (278/421) [62.8, 69.1]	81.2 (643/792) [78.3, 83.8]	-	-
Upgraded LR-5 using malignancy AF in general	88.2 (448/508) [85.1, 90.9]	95.1 (270/284) [91.9, 97.3]	97.0 (448/462) [95.1, 98.2]	81.8 (270/330) [78.0, 85.1]	90.7 (718/792) [88.4, 92.6]	<0.001	0.008
Upgraded LR5 using HCC AF	78.9 (401/508) [75.1, 82.4]	97.5 (277/284) [95.0, 99.0]	98.3 (401/408) [96.5, 99.2]	72.1 (277/384) [68.6, 75.4]	85.6 (678/792) [83.0, 88.0]	<0.001	>0.999
LR-5 after extending the evaluation of APHE to the subtraction image**	74.4 (378/508) [70.4, 78.2]	97.9 (278/284) [95.5, 99.2]	98.4 (378/384) [96.6, 99.3]	68.1 (278/408) [64.8, 71.3]	82.8 (656/792) [80.0, 85.4]	<0.001	>0.999
LR-5 when considering no washout if no APHE.	71.3 (362/508) [67.1, 75.2]	97.9 (278/284) [95.5, 99.2]	98.4 (362/368) [96.5, 99.3]	65.6 (278/424) [62.4, 68.6]	80.8 (640/792) [77.9, 83.5]	0.250	>0.999
LR-5 after extending evaluation of washout from PVP to TP.	75.6 (384/508) [71.6, 79.3]	96.8 (275/284) [94.1, 98.5]	97.7 (384/393) [95.7, 98.8]	68.9 (275/399) [65.5, 72.1]	83.2 (659/792) [80.4, 85.8]	<0.001	0.250
LR-5 if not using subthreshold (subthreshold = threshold) [LR v2017 vs. LR v2018]	74.8 (380/508) [70.8, 78.5]	97.9 (278/284) [95.5, 99.2]	98.5 (380/386) [96.6, 99.3]	68.5 (278/406) [65.1, 71.6]	83.1 (658/792) [80.3, 85.6]	<0.001	>0.999

All diagnostic performances are calculated for HCC.

Abbreviations: AF, ancillary features; APHE, (nonrim) arterial phase enhancement; HCC, hepatocellular carcinoma; LI-RADs, Liver Imaging Reporting and Data Systems; PVP, portal venous phase; TP, transitional phase.

**APHE is evaluated in both the arterial phase image and the subtraction (arterial phase-precontrast phase) image.

Numbers in parentheses are the 95% confidence intervals (CIs).

^*a*^*P*-value after comparing sensitivity to that of LR-5* using McNemar’s test

^*b*^*P*-value after comparing specificity to that of LR-5* using McNemar’s test

### Diagnostic performance of LR-5 for HCC after category adjustment of LR-4 using AFs

Among the total 118 LR-4 observations, 83 observations (70.3%) were found eligible for upgrade to LR-5 using AFs favoring malignancy in general (including AFs favoring HCC in particular) defined by LI-RADS v2018. This adjusted LR-5 resulted in significantly increased sensitivity (88.2%, *P*<0.001) and decreased specificity (95.1%, *P* = 0.008) for HCC (Table 2).

On the contrary, when applying AF favoring HCC in particular only, 36 out of a total 118 LR-4 observations (30.5%) were found eligible for upgrade to LR-5. After category adjustment, LR-5 sensitivity significantly increased (78.9%, *P*<0.001) without decreasing its specificity (97.5%, *P*>0.999) for HCC (Table 2). Comparison of ROCs showed significant increase in AUCs of both LR-5 upgraded with AFs favoring malignancy in general and LR-5 upgraded with AFs favoring HCC in particular (*P*<0.001) (Table 3).

**Table 3:** Area under the curve (AUC) of various categorizations.

	AUC (95% CI)	*P*-value^a^
LIRADS v2018 LR-4 and 5	0.936 [0.917, 0.952]	-
LIRADS v2018 LR-5*	0.849 [0.822, 0.873]	-
Upgraded LR-5 using malignancy AF in general	0.916 [0.895, 0.935]	<0.001
Upgraded LR5 using HCC AF	0.886 [0.862, 0.908]	<0.001
LR-5 after extending the evaluation of APHE to the subtraction image**	0.861 [0.835, 0.885]	<0.001
LR-5 when considering no washout if no APHE.	0.846 [0.819, 0.870]	0.083
LR-5 after extending evaluation of washout from PVP to TP.	0.862 [0.836, 0.885]	0.010
LR-5 if not using subthreshold (subthreshold = threshold) [LR v2017 vs. LR v2018]	0.863 [0.838, 0.885]	<0.001

Numbers in parentheses are the 95% confidence intervals (CIs).

All diagnostic performances are calculated for HCC.

Abbreviations: AF, ancillary features; APHE, (nonrim) arterial phase enhancement; HCC, hepatocellular carcinoma; LI-RADs, Liver Imaging Reporting and Data Systems; PVP, portal venous phase; TP, transitional phase.

^a^*P*-value of pairwise comparison of ROC curves (compared to LR-5*)

### Diagnostic performance of LR-5 for HCC after extending APHE to the subtraction image

**Fig 2:**
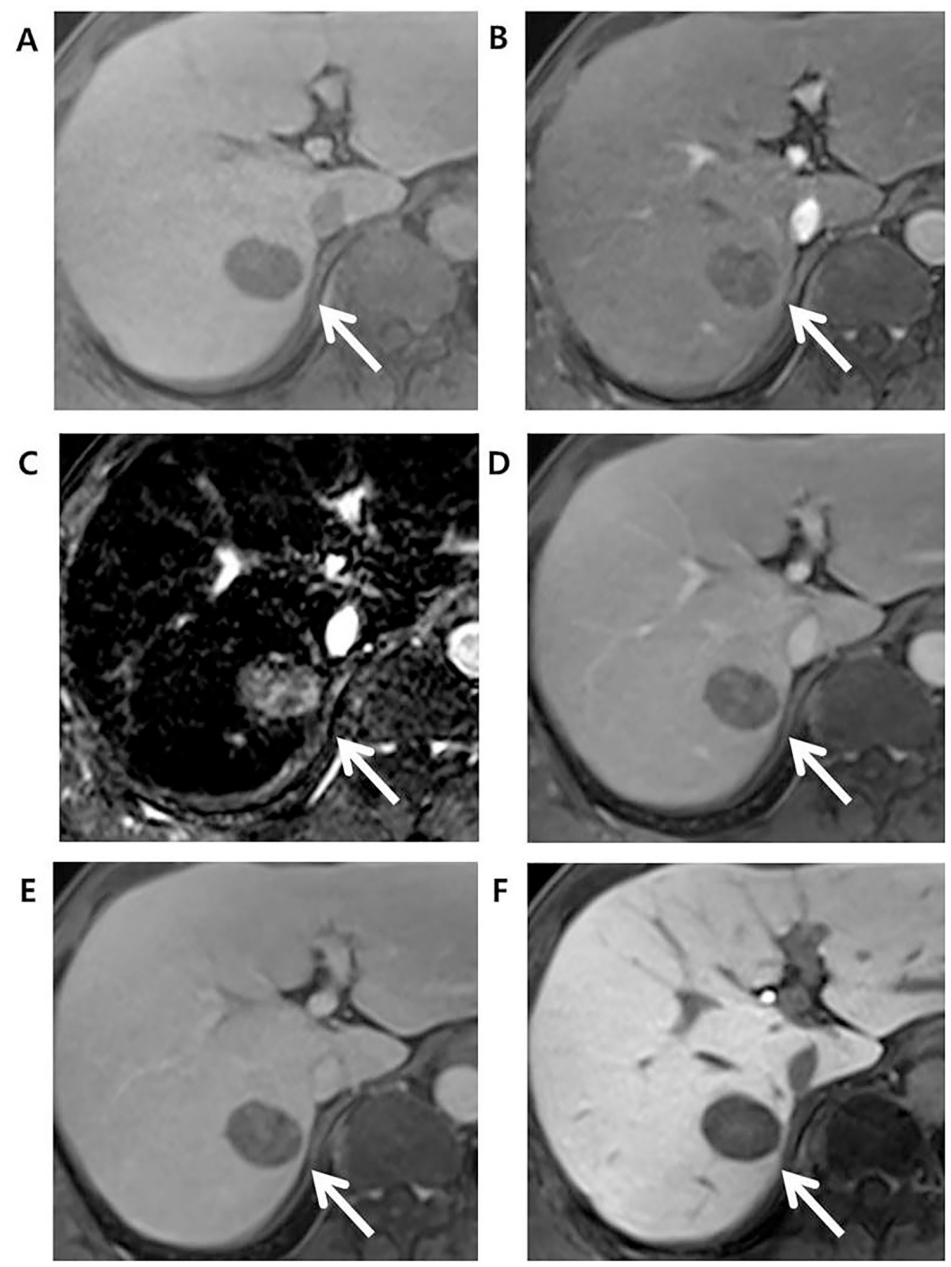
Edmonson grade 3 HCC in 49-year-old male with underlying B-viral chronic hepatitis. Compared to the (A) axial pre-contrast phase, the (B) late arterial phase (AP), and (D) portal venous phase (PVP) images after gadoxetate administration showed a 30mm-sized liver mass (arrow) in segment 7 (S7) of the liver with no nonrim arterial hyperenhancement in the late arterial phase but washout in the portal venous phase. Initially, this hepatic observation was categorized as LR-4. (C) The arterial subtraction image, however, showed homogeneous enhancement compared to the normal liver parenchyma. This hepatic observation was thus recategorized as LR-5 based on the arterial subtraction image. (E) Delayed phase and (F) hepatobiliary (HBP) phase images showed decreased signal intensity in the hepatic observation compared to liver parenchyma.

As for the detection of APHE, among 81 HCCs that did not show APHE in late arterial phase, 16 (19.8%) showed APHE in arterial subtraction images. Out of these 16 observations, 13 observations had their final LR categories adjusted from LR-4 to LR-5 when APHE was interpreted using subtraction image (Fig 2). All 13 observations were later confirmed as HCCs.

Similarly, among 73 LR-M observations, one observations showed iso-to-hypointensity with peripheral rim enhancement in late arterial phase but a homogenous hyperenhancement in the subtraction image. The final LR category of this observation was adjusted to LR-5 from LR-M and was also confirmed as HCC. The adjusted LR-5 showed a sensitivity of 74.4% and a specificity of 97.9% for HCC, and a significant difference was noted in sensitivity (*P*<0.001) but not in specificity (P>0.999) when compared to original LR-5 (Table 2).

#### Diagnostic performance of LR-5 for HCC considering no washout if APHE was absent

Out of 81 HCCs that showed no APHE, 77 (95.1%) HCCs showed washout in portal venous phase. When washout was considered absent because APHE was absent, three LR-5 observations were readjusted to LR-4. Diagnostic performance of LR-5 after this adjustment showed a sensitivity of 71.3% (*P* = 0.250) and a specificity of 97.9% (*P*>0.999).

### Diagnostic performance of LR-5 for HCC after extending washout to the transitional phase image (transitional phase hypointensity as a major feature)

Out of 76 HCCs that did not show washout in portal venous phase, 39 (51.3%) showed hypointensity in the transitional phase. Based on LI-RADS v2018, 20 observations were assigned LR-4, 16 observations were assigned LR-5 and three observations were assigned LR-M. When transitional phase hypointensity was also considered to indicate washout, a major feature, 19 LR-4 observations were readjusted to LR-5, resulting in 1 LR-4 and 35 LR-5. Diagnostic performance of adjusted LR-5 showed a sensitivity of 75.6% (*P*<0.001) and a specificity of 96.8% (*P* = 0.250) (Table 2).

### Diagnostic performance of LR-5 for HCC when subthreshold growth was considered a major feature similar to threshold growth in LI-RADS v2017

Among the total 551 LR-3, -4, and -5 observations, 34 observations (6.2%) showed subthreshold growth wherein 30 of these 34 observations (88.2%) were histopathologically confirmed as HCC. Initially, the final LR-categories of the 34 observations were LR-4 for 26 observations and LR-5 for 8 observations. However, when subthreshold growth was regarded as a major feature as it was in LI-RADS v2017, 15 LR-4 observations were readjusted to LR-5. This category-adjusted LR-5 showed a sensitivity of 74.8% and a specificity of 97.9% (Table 2). Compared to original LR-5, significant increase in sensitivity (*P*<0.001) and non-significant decrease in specificity (*P*>0.999) were noted.

## Discussion

Our results indicate that compared to the diagnostic performance of LR-5 based on LI-RADS v2018, category-readjusted LR-5 after upgrading LR-4 to LR-5 using AF favoring HCC in particular, subthreshold growth as a major feature, extending washout to transitional phase and APHE interpreted using arterial subtraction images can significantly increase sensitivity without reducing specificity for HCC. On the other hand, LR-5 upgraded from LR-4 using AF favoring malignancy in general showed significant decrease in specificity for HCC despite increased sensitivity. With washout being considered when APHE was absent, there were no significant changes in either sensitivity or specificity of LR-5 for HCC.

When categories were adjusted using AFs, upgrading LR-4 to LR-5 with AF favoring malignancy in general was found to significantly decrease the specificity of LR-5 for HCC because most of the LR-4 lesions showed at least one AF favoring malignancy. This finding is consistent with an explanation given by LI-RADS v2018 [7] where it states that AFs do not show sufficient specificity for HCC. While there have been previous studies demonstrating that AFs favoring malignancy in general show high specificity for HCC [4, 12], readjusted LR-5 after applying these features failed to increase specificity. However, aligned with our expectations, LR-5 readjusted after using AF favoring HCC in particular showed no significant reduction in specificity for HCC while increasing the sensitivity for HCC

**Fig 3:**
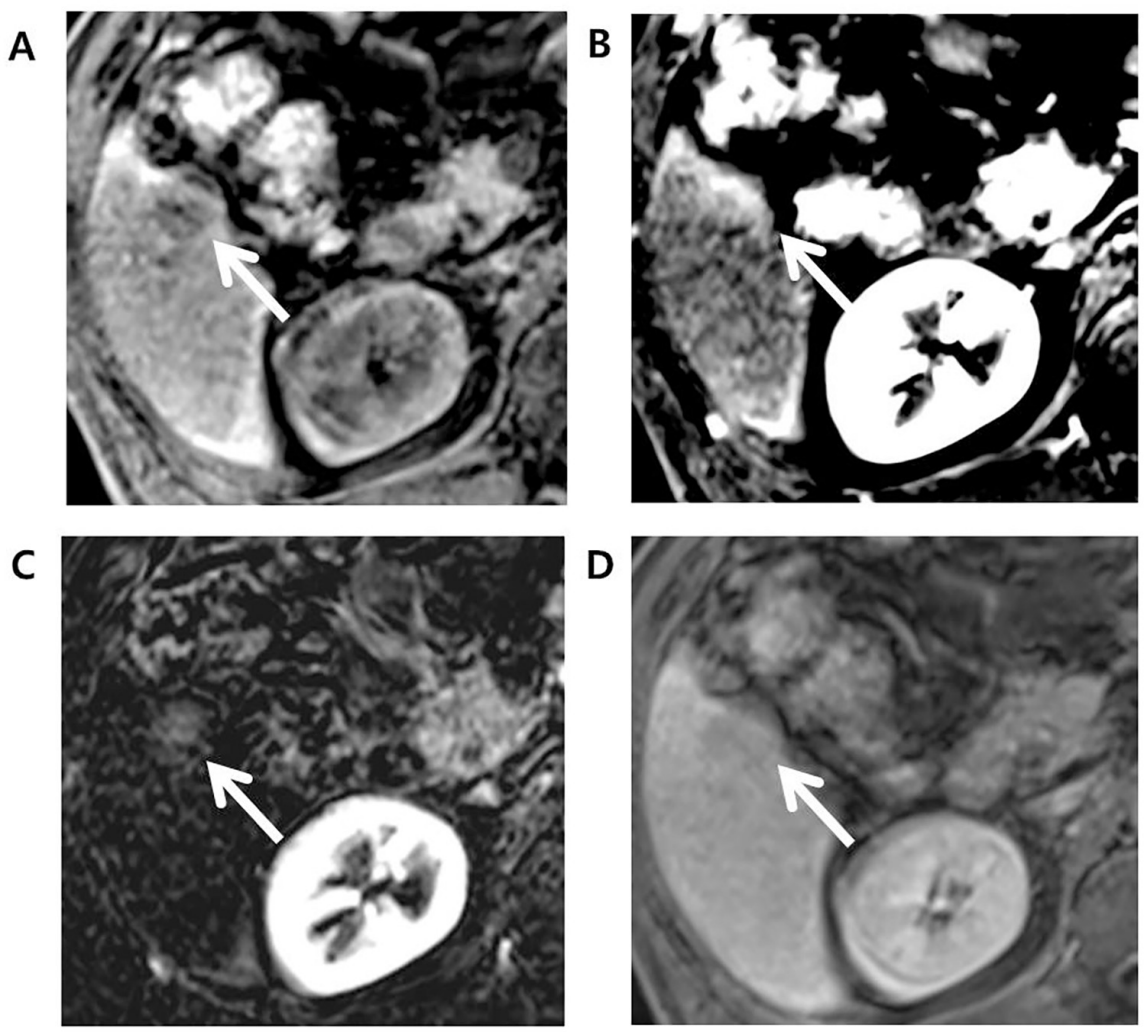
Edmonson grade 1 HCC in 71-year-old male with underlying hepatitis B-viral chronic hepatitis. (A) Axial pre-contrast phase shows a 23mm sized liver mass (arrow) in segment 6 (S6) of the liver. (B) Late arterial phase (AP) shows rim-like arterial hyperenhancement and thus, this observation was categorized as LR-M considering targetoid appearance. (C) Arterial subtraction image, however, shows a global homogeneous enhancement and, with presence of threshold growth, this observation was re-categorized as LR-5. (D) Delayed phase shows hypointensity compared to background liver.

As for category adjustment after extending APHE to the arterial phase subtraction images, adjusted LR-5 showed increased sensitivity without decreasing the specificity for HCC. This result was consistent with a recent study [13], thereby validating that the use of subtraction image can contribute to the diagnostic accuracy of LR-5 observations. Furthermore, one observation showed rim-like enhancement pattern in arterial phase and was categorized initially as LR-M. However, the subtraction image was helpful in confirming a global enhancement pattern, which with threshold growth and size criterion, recategorized the observation as LR-5 (Fig 3). This observation was later confirmed as HCC, demonstrating that subtraction image can help facilitate categorization of false-positive LR-M observation back to LR-5. Possible explanation is that in case where the tumor center and periphery show enhancement compared to background liver but the tumor center shows slightly weaker enhancement than periphery, this difference in the enhancement degree is eccentuated in late arterial phase and an observation may be mistaken to exhibit rim-like enhancement pattern. However, this difference is less prominant in subtraction image, where both tumor center and periphery show increased intensity when precontrast scan is subtracted from arterial phase, thus showing a global enhancement pattern, which the reader can use to correctly categorize an observation as LR-4 or 5.

LR-5 adjusted while considering no washout when APHE was absent in the artertial phase failed to show any significant change in both sensitivity and specificity for HCC, with the sensitivity for HCC rather showing a slight decrease compared to that of the original LR-5. While there is a debate as to whether washout should be considered at all when APHE is absent, associating washout to APHE allowed LR-5 observations to only downgrade to LR-4 by reducing a major feature. Our result thus indicates that washout should be considered separately from APHE as suggested by the current LI-RADS v2018.

Lastly, we evaluated the diagnostic performance of LR-5 when subthreshold growth is considered a major feature. While only 6.2% of all non-LR-M observations showed category readjustment after applying subthreshold growth as a major feature (i.e. applying the threshold growth criteria of LIRAD v2017), separating subthreshold growth from threshold growth as in LI-RADS v2018 significantly decreased LR-5 sensitivity for HCC, even though no significant reduction was noted for LR-5 specificity. By removing subthreshold growth from major features, some LR-5 observations were recategorized as LR-4 due to the loss of a major feature and this explains for the lower LR-5 sensitivity for HCC.

This study has several limitations. First, the present study may have a selection bias due to its single-center retrospective nature even though we tried our best to minimize this limitation by including a large number of study patients. Secondly, image analysis was performed by two radiologists in consensus, and thus, no interobserver agreement could be determined. Thirdly, all patients underwent gadoxetate disodium MRI, which is known to show more ghosting artifact in arterial phase known as transient severe motion artifact than extracellular agent (ECA)-based MRI but we did not encounter difficulty during image analysis as patients underwent MRI re-examination when transient severe motion artifact impeded image analysis.

In conclusion, upgrading LR-4 to LR-5 using AF favoring HCC in particular, using subthreshold growth as a major feature, extending washout to transitional phase, and interpreting APHE using arterial subtraction image significantly increased the sensitivity of LR-5 for HCC without significantly reducing the specificity.
